# Transformer model generated bacteriophage genomes are compositionally distinct from natural sequences

**DOI:** 10.1093/nargab/lqae129

**Published:** 2024-09-18

**Authors:** Jeremy Ratcliff

**Affiliations:** Johns Hopkins University Applied Physics Laboratory, 11000 Johns Hopkins Road, 20723 Maryland, Laurel, MD 20723, USA

## Abstract

Novel applications of language models in genomics promise to have a large impact on the field. The megaDNA model is the first publicly available generative model for creating synthetic viral genomes. To evaluate megaDNA’s ability to recapitulate the nonrandom genome composition of viruses and assess whether synthetic genomes can be algorithmically detected, compositional metrics for 4969 natural bacteriophage genomes and 1002 *de novo* synthetic bacteriophage genomes were compared. Transformer-generated sequences had varied but realistic genome lengths, and 58% were classified as viral by geNomad. However, the sequences demonstrated consistent differences in various compositional metrics when compared to natural bacteriophage genomes by rank-sum tests and principal component analyses. A simple neural network trained to detect transformer-generated sequences on global compositional metrics alone displayed a median sensitivity of 93.0% and specificity of 97.9% (*n* = 12 independent models). Overall, these results demonstrate that megaDNA does not yet generate bacteriophage genomes with realistic compositional biases and that genome composition is a reliable method for detecting sequences generated by this model. While the results are specific to the megaDNA model, the evaluated framework described here could be applied to any generative model for genomic sequences.

## Introduction

The widespread development and application of language models has expanded their impact beyond traditional application in natural language processing. Transformer models, which leverage self-attention mechanisms and deep neural networks, are able to learn the intricate patterns and implicit rules of language. Genomics is essentially a form of language, with nucleotide (and protein) sequences encoding biological information based on the content and order of characters. Transformer-based genomic language models have proven to be particularly adept at identifying ‘missing’ annotations for nucleotide sequence data, including for promoters, splice sites, enhancers, chromatin accessibility, transcription factor binding sites and more (1–5). Less common, however, has been the application of transformer-based models for generating novel nucleotide sequences.

In December 2023, Shao published the first version of their language model ‘megaDNA’ for the generation of *de novo* synthetic bacteriophage genomes (6). The megaDNA model leverages the architecture underlying the MEGABYTE model from Meta AI, which is specifically designed for long input sequences (7). In the preprint, Shao used a random four-base pair (bp) primer to generate 1024 sequences longer than 1 kilobase (kb) and demonstrated that a portion of these sequences can be classified as viruses by an artificial intelligence/machine learning (AI/ML)-based classification framework, showing realistic gene lengths and distributions, and encode potentially functional 5′ untranslated regions, genes and promoters.

Genome composition is the reduction of nucleotide sequences into quantitative descriptions of the frequency of specific patterns. These measures can be used to assess the nonrandom distribution of nucleotides in a genome, including measures such as guanine and cytosine (GC) content, di- and trinucleotide odds ratios, codon pair bias and secondary structure elements, among others. RNA viruses have been extensively studied for compositional biases, showing family- and host-dependent patterns (8,9). While less thoroughly studied, bacteriophages have also shown diversity in GC content and select dinucleotide ratios (10). These biases, manifesting in genome-level measurements, were hypothesized to be an appropriate benchmark for megaDNA’s ability to recapitulate higher-order relationships. Identification of a computational method to discriminate transformer-generated from natural sequences would have broad applicability to synthetic biology, biosecurity and future applications of AI/ML to genomics.

Here, compositional biases in sequences generated by megaDNA were compared to those of natural bacteriophage sequences from the NCBI RefSeq database. Using a variety of approaches, these sequences are shown to be compositionally distinct from the natural sequences upon which they were trained. A variety of methods of varying complexity, from single metric rank-sum tests to 19-feature neural networks, differentiate the two populations with high accuracy on the basis of quantitative compositional metrics alone.

## Materials and methods

### Creation of transformer-generated sequences

Transformer-generated sequences were produced using the model and model inference codes from (6), as of 27 December 2023: https://github.com/lingxusb/megaDNA. The inference code was modified to encapsulate the model.generate() call within a with torch.no_grad() statement to reduce memory consumption. Model inference was performed on an NVIDIA V100.

### Identification of natural bacteriophage genomes

Shao (6) does not list the accession numbers for all sequences used to train the transformer model described in the preprint. To ensure that only high-confidence and high-quality sequences of real bacteriophages were used for comparison, *n* = 4984 RefSeq sequences containing ‘phage’ within the organism’s name were identified from the NCBI virus database in December 2023. There is a high likelihood these sequences are present within the training set used by Shao (6). It is currently unknown whether the training data used by megaDNA are as or more diverse than the sequences used in this study, although this information may become available with revisions to the megaDNA manuscript. GenBank files for each accession number were downloaded from NCBI using the Entrez API available via Biopython (11).

### Sequence compositional analysis

Sequence composition analysis was performed using custom Python scripts. GC content and dinucleotide odds ratios were calculated for whole genomes. Structural metrics [minimum free energy (MFE) and minimum free energy difference (MFED)] were calculated for overlapping subsequences for each genome from which median values were extracted. Briefly, a sequence was exploded into overlapping *k*-mers of prespecified length and step size and stored in a numpy array (12). Metrics were calculated for each *k*-mer and values assigned to the index of the midpoint nucleotide of the *k*-mer in the original sequence. For this study, a *k*-mer size of 120 bp and a step size of 10 bp were used.

#### GC content

GC content was calculated as the sum of guanine and cytosine bases in the sequence divided by the length of the sequence.

#### Dinucleotide odds ratios

Dinucleotide odds ratios were calculated based on the expectation of a random assortment of mononucleotides of fixed frequencies. Therefore, ratios of observed versus expected dinucleotide frequencies can be derived for any sequence with a known mononucleotide frequency. Odds ratios <1 one represent suppression, while ratios >1 indicate overexpression.

Formally, the dinucleotide ratio for any two nucleotides, X and Y, can be calculated as


\begin{eqnarray*} \frac{Observed}{Expected} = \frac{f(XpY)}{f(X)*f(Y)}, \end{eqnarray*}

where *f*(XpY) is the observed frequency of dinucleotide XpY, *f*(X) is the observed mononucleotide frequency for mononucleotide X and *f*(Y) is the observed mononucleotide frequency of Y.

#### Minimum free energy

MFE values were calculated using the RNA.fold() function from the Vienna RNA Python package (13). While designed for single-stranded (ss) RNA, these algorithms were applied equally to ss and double-stranded (ds) RNA and DNA sequences. See the next section for a disclaimer about deriving structural metrics for dsDNA genomes.

#### Minimum free energy difference

MFED values were calculated as the percentage difference between the MFE of a sequence and those of a selection of permuted controls, as performed elsewhere (14). There are multiple approaches for creating permuted controls that can lead to inconsistent conclusions (15). For every 120-bp oligomer, a dinucleotide shuffling algorithm was used to generate a set of 105 permuted controls against which the MFE of the oligomer was compared (see Supplementary Methods and Supplementary Figure S6). *Z* scores for MFED values were calculated by comparing the observed MFE value (*X*) to the mean (*μ*) and standard deviation (*σ*) of MFE values of the permuted controls:


\begin{eqnarray*} Z = \frac{X-\mu }{\sigma }. \end{eqnarray*}

Of note, Shao describes 98% of the sequences used in the model training dataset as being members of the *Caudovirales* class of dsDNA phages. As these are expected to be fully base paired, it is reasonable to question the use of MFE or MFED values to describe their composition. However, the natural genome sequences used in this study, when treated as ssRNA, were consistently more structured than would be expected by chance (97.6% with median values >0 (*n* = 4850/4969); *P* < 2.2e−308, binomial distribution). This result confirms that MFE and MFED results can be used as measurements of the nonrandomness of bacteriophage base composition even if their contribution to nucleic acid binding dynamics is unclear.

#### Dinucleotide-shuffled sequence generation

Dinucleotide shuffling is a method of shuffling sequences in a manner that conserves the nonrandom distribution of dinucleotide frequencies of the input sequences. Here, dinucleotide shuffling was conducted using an open-source implementation of the Altschul–Erikson algorithm written in Python (16): https://github.com/wassermanlab/BiasAway/.

### Gene prediction

Putative genes were identified from transformer-generated and natural sequences using PHANOTATE v1.5.0 (17). PHANOTATE was preferred over other gene callers as it is tuned to the challenges of gene prediction in bacteriophage genomes.

### Virus score prediction

All sequences were processed using geNomad v1.7.4 with the -- relaxed parameter and the MMseqs2 release 15-6f452 (18,19).

### Principal component analysis

Principal component analysis (PCA) was conducted on scaled compositional metrics using the prcomp function in R version 4.2.0 (20). Group centroids were calculated as the mean value of each PC and expressed as a matrix of coordinates. The ‘distance’ between points or group centroids in the 19-dimensional space was calculated as a Euclidean distance measure weighed based on the square of each principal component’s (PC) eigenvalue. Weighed Euclidean distance was used in place of downselecting PCs using the Kaiser criterion or a scree plot due to criticism of the two methods being arbitrary or subjective (21,22). Further, Euclidean distance was preferred to Mahalanobis distance as the raw data were scaled to unit variance before the PCA was performed. As the square of the eigenvalue is equivalent to the proportion of variance explained by each PC, distance measures are dominated by the PCs that explain the greatest proportion of the total variance.

Nearest neighbors for individual points were identified using function get.knn from the FNN package. The null hypothesis of random association predicts that the frequency of a nearest neighbor being from the same family of a given point is equal to


\begin{eqnarray*} \frac{N_f}{N}, \end{eqnarray*}

where *N*_*f*_ is the total number of points for family *f* and *N* is the total number of points in the dataset.

### Predictive neural network

To create a predictive neural network in Python, compositional metric data were loaded into a pandas DataFrame, and all features were scaled using StandardScaler() from scikit-learn (23). Sequence provenance (natural versus transformer) was encoded as 0 or 1, respectively, and an 80:20 training:test split was used. The neural network architecture used the Sequential() model from the TensorFlow Keras API (24). The model consisted of two hidden layers with 32 and 16 neurons, respectively, both with rectified linear unit activation functions, and an output layer with 1 neuron and a sigmoid activation function. The model was compiled using binary cross-entropy loss and the Adam optimizer and trained using 10 epochs and a batch size of 32. Model performance on the testing data was expressed as total accuracy, sensitivity for transformer-generated sequences and specificity for natural sequences. The training:test split was not altered during model evaluation or the predictive feature selection—that is, to say, identical sequences were labeled as training or test data for each iteration of model training and evaluation.

### Predictive feature selection

Predictive feature evaluation was performed iteratively to maximize (or minimize) the predictive capability of models with limited features. This iterative approach began with a baseline model containing no features. During each step, each of the remaining features was added to the existing set of features and used to train 12 independent models. Following the evaluation of each feature, the metric whose addition resulted in the highest (or lowest) average increase in accuracy was selected and incorporated into the model. This partial model was used to continue the iterative process until all features had been selected.

### Statistical analysis

Between-group comparisons were made using two-tailed Mann–Whitney tests. Binomial distributions were used in instances where binary outcomes could be derived (e.g., *k*-nearest neighbor analysis of family clustering, order of relationships within geNomad, etc.).

## Results

### Transformer-generated sequence composition is generally dissimilar to that of natural sequences

To compare the composition of natural and transformer-generated bacteriophage genomes, 4969 RefSeq bacteriophage genomes were downloaded from NCBI and 1095 synthetic genomes were produced using the megaDNA model with a random 4-bp primer. These synthetic sequences are referred to as ‘transformer-generated’. Consistent with (6), analyses were limited to the 1002 transformer-generated sequences that were greater than 1 kb in length. In contrast to (6), however, natural sequences with lengths greater than 96 kb were not removed. These longer sequences (*n* = 721) represent 14.4% of the natural composition dataset.

**Figure 1.**
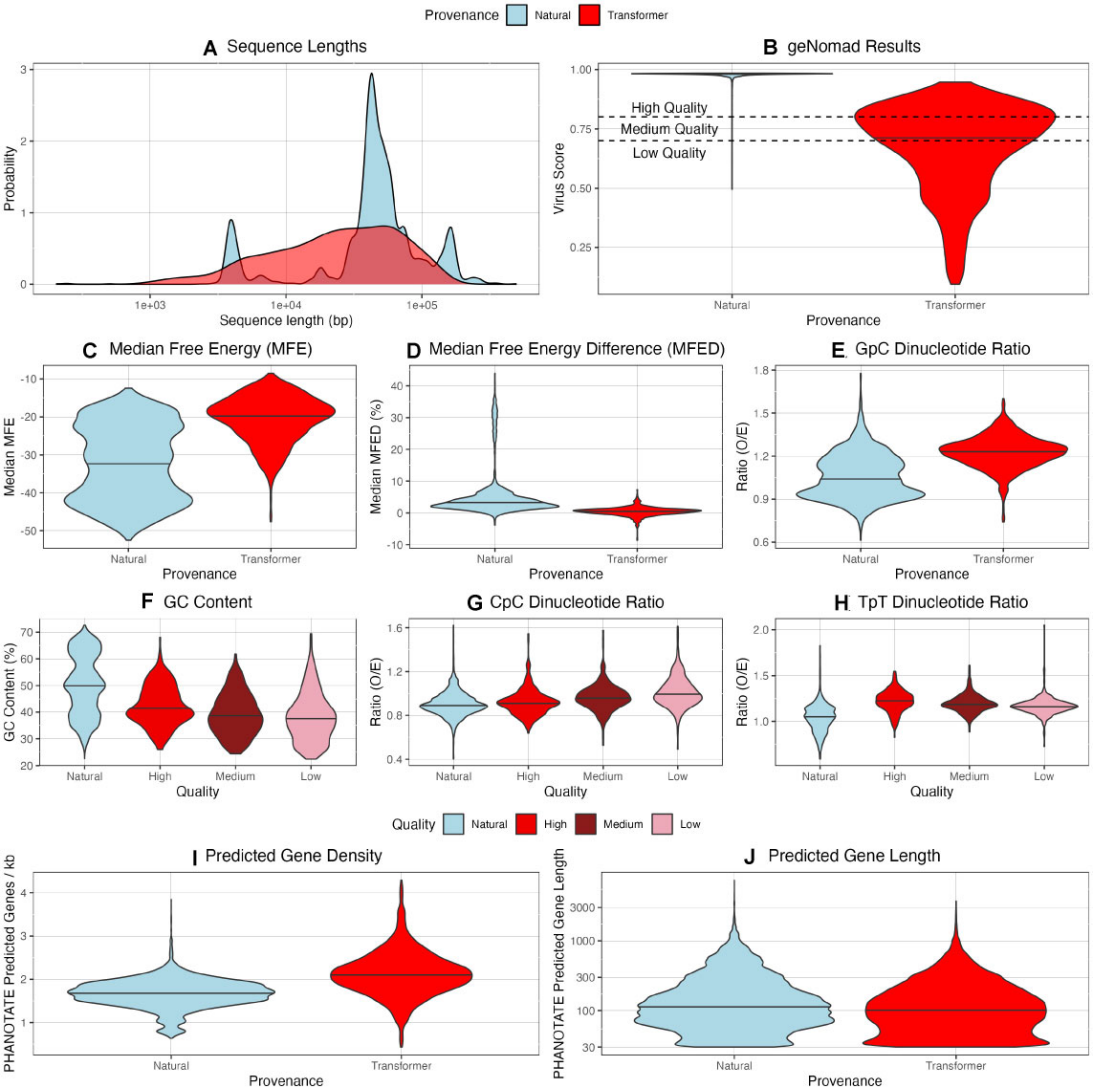
Characteristics of natural and transformer-generated sequences. For all violin plots, the black horizontal bar displays the median value. (**A**) Sequence length for natural sequences and all transformer-generated sequences ≥1000 bp. Probability distributions significantly different (*P* < 2.2e−308, two-tailed Kolmogorov–Smirnov test). (**B**) geNomad virus score results. Quality cutoffs of 0.7 and 0.8 are based on parameters described within the software documentation. (**C–E**) Differences between select metrics; all distributions significantly different by two-tailed Mann–Whitney *U* tests (*P* < 0.0026). (**F–H**) Differences between select metrics with transformer-generated sequences grouped according to geNomad virus quality; GC content and TpT ratio significantly different between natural and high-quality sequences (*P* < 0.0026). (**I, J**) Characteristics of genes predicted by PHANOTATE. Both distributions are significantly different by two-tailed Mann–Whitney *U* tests.

Natural sequences had a median length of 46 kb, with noted peaks around 6, 40 and 170 kb (Figure 1A). Transformer-generated sequences were on average shorter (median length of 23.7 kb) and displayed a much smoother probability distribution. Length distributions were significantly different (*P* < 2.2e−308, two-tailed Kolmogorov–Smirnov test). The smooth distribution of the transformer-generated sequences is not unexpected, as the predictive approach of megaDNA can infer that the random 4-bp primer sequence is located at any position in a theoretical genome. Thus, the sequence output can essentially be thought of as fragments that always extend to the 3′ end of the genome.

geNomad, an AI/ML-enabled taxonomic classification framework, was used to assess the ability of megaDNA to produce sequences classified as viral by non-*k*-mer approaches (18). As expected, transformer-generated sequences produced significantly lower virus scores than the natural sequences (Figure 1B; median values of 0.72 and 0.98, respectively, *P* < 2.2e−308, two-tailed Mann–Whitney *U* test). Only 22 transformer-generated sequences had predicted taxonomy, all of which were *Caudoviricetes*. Based on geNomad virus scores, transformer-generated sequences were split into low (virus score <0.7; *n* = 418), medium (virus score ≥0.7 and <0.8; *n* = 281) and high quality (virus score ≥0.8; *n* = 303). In this study, geNomad was executed with the -- relaxed quality parameter to ensure scores were reported for all sequences. This explains the on average higher geNomad score results reported in (6), where geNomad was almost certainly executed with default quality parameters. The cause of the much lower classification efficiency in this study compared to (6) is unclear but could be due to using different versions of geNomad or MMSeqs2. The version numbers were not specified in (6).

Nineteen compositional metrics were analyzed. These included GC content, odds ratios for all 16 dinucleotides, median MFE values for overlapping 120-bp subsequences and MFED values calculated against dinucleotide-shuffled controls. Of the 19 compositional metrics considered, the distributions for natural versus transformer-generated were significantly different for 18 of the metrics after a Bonferroni correction (examples in Figure 1C–E; Supplementary Figure S1). For dinucleotide odds ratios, absolute differences in medians ranged from 0.0003 (ApA) to 0.194 (GpC). Transformer-generated sequences were consistently more GC-poor (median values of 39.2% versus 49.9%), had lower folding energy (median MFE values of −19.7 versus −32.2) and had MFED values much closer to the null expectation (0.005 versus 0.0324) than natural sequences. Differences in MFED values were particularly stark, as only 3% of transformer-generated sequences displayed MFED values as high as the median natural sequence (Figure 1D). The median standard deviation for MFED values from transformer-generated sequences was slightly but significantly higher than that for natural sequences (medians of 0.172 versus 0.150, *P* = 8.78e-43), suggesting that the variability in MFED values for transformer-generated sequences may contain some information regarding how this model has ‘learned’ interactions in near-adjacent bases. For both natural and transformer-generated sequences, there are short segments with sustained levels of higher MFED values (Supplementary Figure S2A and B). The selection of 120 bp as the subsequence window size may be slightly fortuitous, as the degree of structure compared to chance (measured by *Z* score) was significantly lower for both natural and transformer-generated sequences when *Z* scores were calculated using 85 bp (median values of −0.29 versus −0.19 and −0.036 versus −0.0045, respectively, *P* < 2.2e−308 and *P* = 2.09e−74, paired Mann–Whitney *U* tests; Supplementary Figure S2C and D).

The neural network classification module in geNomad uses 4-mer encoded subsequences, which may lead to scores reflecting compositional differences. Supporting this, 72% of comparisons of composition metrics between low-, medium- and high-quality transformer-generated sequences were significantly different (Supplementary Table S1). If the geNomad scores were implicitly detecting composition bias toward natural sequences, it would be expected that high-quality sequences would be most similar to natural, low quality would be the most different and medium quality would be somewhere in the middle (as in Figure 1F). Indeed, this pattern was observed for 36.8% of compositional metrics, significantly more frequently than would be expected by chance (*P* = 7.6e−5, binomial distribution). However, these high-quality transformer-generated sequences still had significantly different composition metric distributions than natural sequences for 17 of the 19 measured metrics (Figure 1F–H; Supplementary Table S1).

Differences in nucleotide composition may also present as variation in encoded information. To assess any differences in gene content, PHANOTATE was used to predict genes in both natural and transformer-generated sequences. PHANOTATE has advantages over other gene prediction algorithms for this project as it is designed for phage genomes, including allowing for multiple start codons and limiting strand switching (17). Using PHANOTATE outputs, transformer-generated sequences had a significantly higher number of predicted genes per kb than natural sequences (median values of 2.10 and 1.68, *P* = 7.28e−185, two-tailed Mann–Whitney *U* test; Figure 1I). Predicted genes for transformer-generated sequences were also slightly but significantly shorter (median values of 100 and 113, *P* < 2.2e−308, two-tailed Mann–Whitney *U* test; Figure 1J). As these metrics were derived from a predictive algorithm for which this project did not seek to perform validation, they were not incorporated into downstream PCA or neural network analysis. Minor functional characterization of predicted genes is described in the Supplementary Results.

Overall, the transformer-generated sequences display significantly altered composition metrics when compared to the population of RefSeq sequences identified from NCBI, which may or may not reflect the true diversity included in the megaDNA training data. Additionally, a portion of the sequences are classified as viral by geNomad, and there is some evidence of a relationship between virus score and ‘natural-like’ composition.

### Bacteriophage sequences cluster based on provenance and taxonomic relationships

It is generally observed that genome composition varies between viral families (9). Without knowledge as to the specific sequences used to train megaDNA, it is possible that the training sequences were biased toward an individual family, and, as such, comparisons against a population of bacteriophage sequences that do not reflect the same bias—as done in the previous section—are unfair and harsh. To evaluate whether transformer-generated sequences are simply many members of a specific viral family to which the model is overfitting, PCA of the compositional metrics was performed. PCA was preferred to the use of raw metrics as the nature of dinucleotide ratios leads to many correlations between them (Supplementary Figure S3).

**Figure 2.**
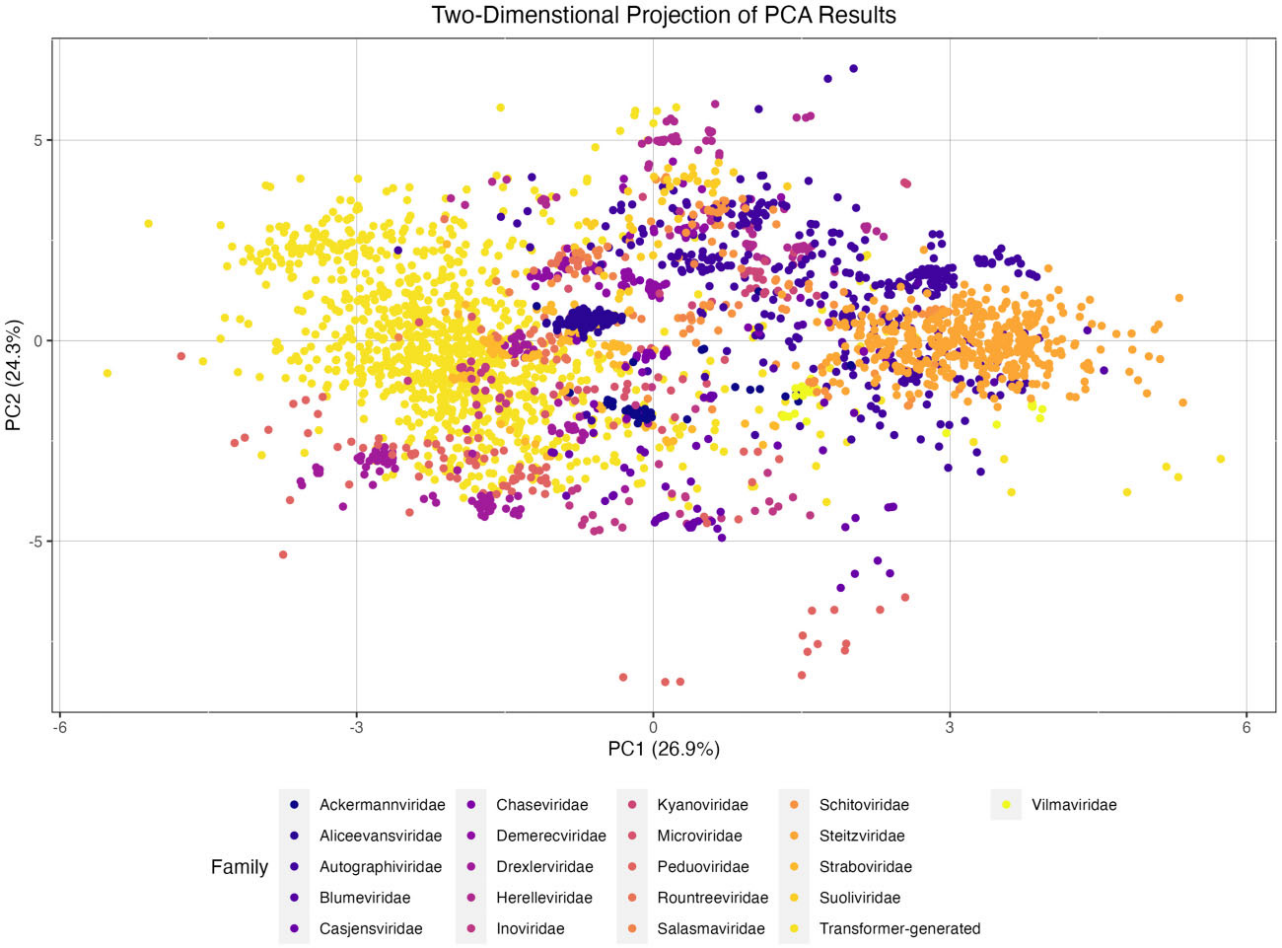
PCA of compositional metrics. Two-dimensional projections of PCs 1 and 2 limited to sequences from families with ≥25 members, colored by taxonomy. The values for this projection have not been weighted according to the square of the eigenvalues, although they are for any distance calculations. Percentages in *x*-axis and *y*-axis titles indicate percentage of variance explained by given PC. Kindly note that the two-dimensional projection can be misleading as to the true location of a data point in the 19-dimensional space created by the PCA.

PCA was performed using data for all sequences. For cluster and distance analysis and visualization, however, the comparisons were limited to only transformer-generated sequences and those bacteriophage genomes whose GenBank file specified the viral family (*n* = 2080) (Figure 2). To determine an individual point’s location in the 19-dimensional space returned by the PCA, the raw PC values were weighted according to the square of their respective eigenvalues (colloquially, the percent of variance explained by that PC). To evaluate the extent of clustering for the 28 families with ≥10 members in the dataset (including transformer-generated as a family), the frequency with which a data point’s nearest neighbor in the weighted 19-dimensional space was a member of the same family was compared to expectations from random associations. For all families, individual members were more likely to be ‘close’ to other members of the same family than random sequences in the dataset (Supplementary Table S2). While all significant by binomial distributions (*P* < 0.002), the strength of clustering varied between families. For *Blumeviridae*, only 5 of 34 members clustered. For *Demerecviridae*, all 84 members of the family clustered with another member of that family, likely due to a combination of the family’s low CpG and high TpA ratios compared to all other sequences (0.82 versus 1.00 and 0.96 versus 0.77, respectively, *P* = 6.89e−20 and *P* = 1.99e−41, two-tailed Mann–Whitney *U* tests). Transformer-generated sequences clustered at a rate of 95.2% (*n* = 954/1002). This result was also demonstrated in a PCA that only included dinucleotide ratios and GC content (Supplementary Figure S4). There were no differences in cluster rate between low-, medium- and high-quality transformer-generated sequences (93.1%, 96.4% and 97.0%, respectively). These results confirmed that PCA extracted family-specific compositional traits from the natural sequences.

To assess which viral family transformer-generated sequences were most similar, a weighted Euclidean distance was calculated between centroids of each viral family that contained ≥25 members. The unitless distance measures from the centroid of the transformer-generated family ranged from 0.24 (*Straboviridae*) to 1.40 (*Steitzviridae*) (Table 1). Of important note, the ‘closeness’ of *Straboviridae* and *Demerecviridae* to the transformer-generated sequences in the 19-dimensional space is especially surprising, as these particular sequences from these families should not be present in the training set used for megaDNA as they are globally longer than the stated 96-kb size exclusion threshold. Further, there were still significant differences between *Straboviridae* and transformer-generated sequences for 12 of the 19 metrics (*P* < 0.0026, two-tailed Mann–Whitney *U* test), strongly suggesting that transformer-generated sequences are not simply generating sequences with the same compositional bias as their most similar family. Transformer-generated sequences were no more distinct from natural phage families as other natural families were to each other (Table 1). For the 48 transformer-generated sequences for which another transformer-generated sequence was not the nearest neighbor, both *Drexlerviridae* (*n* = 10) and *Casjensviridae* (*n* = 4) were over-represented as nearest neighbors (*P* = 6.19e−5 and *P* = 0.002, respectively, binomial distribution). These families are generally, however, not tightly clustered based on distribution statistics from their family centroid (Table 1), increasing the likelihood that these associations are due to spurious chance rather than genuine biological phenomena. *Casjensviridae* were additionally one of the more distinct families within the PCA as measured by the cumulative distance to other centroids (Table 1), having the second largest value of all families with 25 or more members (*n* = 21).

**Table 1.** Euclidean ‘distance’ measurements from group centroids Table limited to families with ≥25 members. Centroids derived as described in methods. Distance from transformer-generated centroid calculated from centroid of family while distribution of distances from within-family centroids calculated using individual genomes. Cumulative distance from other centroids is the sum of distances to centroids of all other families

Family	Total members	Euclidean ‘distance’ from centroid of				Cumulative ‘distance’
		Transformer-generated	Within-family			from other centroids
		value	10th percentile	Median	90th percentile	
Ackermannviridae	50	0.64	0.14	0.14	0.41	14.37
Aliceevansviridae	93	0.41	0.04	0.06	0.10	13.37
Autographiviridae	352	1.11	0.24	0.45	0.79	16.86
Blumeviridae	34	1.28	0.11	0.25	0.46	19.08
Casjensviridae	53	1.12	0.21	0.34	0.67	21.69
Chaseviridae	30	0.71	0.09	0.34	0.49	13.99
Demerecviridae	84	0.70	0.13	0.20	0.34	15.65
Drexlerviridae	108	0.65	0.23	0.33	0.65	20.68
Herelleviridae	95	1.17	0.24	0.39	0.53	21.55
Inoviridae	55	0.71	0.40	0.54	0.72	17.44
Kyanoviridae	37	1.00	0.07	0.16	0.49	16.13
Microviridae	30	0.49	0.16	0.30	0.51	13.87
Peduoviridae	92	0.87	0.12	0.36	1.24	22.16
Rountreeviridae	38	0.44	0.25	0.32	0.38	14.94
Salasmaviridae	29	0.61	0.20	0.30	0.40	14.56
Schitoviridae	94	0.98	0.29	0.51	0.77	15.34
Steitzviridae	412	1.40	0.14	0.24	0.40	20.88
Straboviridae	154	0.24	0.14	0.20	0.53	14.16
Suoliviridae	36	1.01	0.16	0.23	0.42	19.73
Transformer-generated	1002		0.20	0.44	0.85	16.67
Vilmaviridae	28	1.14	0.18	0.24	0.59	17.82^a^

^a^Table limited to families with ≥25 members. Centroids derived as described in the ‘Materials and methods’ section. Distance from transformer-generated centroid calculated from centroid of family, while distribution of distances from within-family centroids calculated using individual genomes. Cumulative distance from other centroids is the sum of distances to centroids of all other families.

Altogether, the PCA results suggest transformer-generated sequences occupy an independent compositional niche. This niche likely reflects the average of the compositional biases of the sequences within the training set and suggests that sequences produced by megaDNA revert to their compositional mean rather than reflecting the compositional bias of a single sequence or family in the training set. This hypothesis could be further assessed by priming megaDNA with fragments of sequences of known taxonomy and assessing the degree to which the produced sequence diverges from the ground truth fragment not seen by the model. By intentionally using sequence fragments directly upstream of classes of genes of interest, there may also be an opportunity to evaluate whether megaDNA can more readily recapitulate well-conserved genes than ones subject to host selection.

### A simple neural network differentiates transformer-generated and natural sequences with very high accuracy

If transformer-generated sequences truly occupied an isolated compositional niche, then they should be able to be identified on the basis of compositional metrics alone. To test this hypothesis, a neural network with a relatively simple architecture of two hidden layers was trained to differentiate natural and transformer-generated sequences based on compositional metrics. Twelve models were generated on an identical 80:20 train:test population using all 19 compositional metrics. These ‘total’ models displayed a median overall accuracy of 97.0% (sensitivity = 93.0%, specificity 97.9%). The consistent training:test split enabled interrogation of whether the frequency with which a transformer-generated sequence was predicted to be natural was related to its geNomad virus score. In contrast to the trend implied by Figure 1F–H, there was no relationship between misidentification rate and virus score by simple linear regression (*P* = 0.30), nor were the distributions of those with ≥1 misidentification (*n* = 32) significantly different than those that were classified correctly all 13 times (*n* = 176; *P* = 0.44, two-tailed Mann–Whitney *U* test). Importantly, the neural network input dataset reflected the natural versus transformer-generated sequence imbalance seen throughout this study. This asymmetry leads to a ZeroR benchmark, where all inputs are labeled the most common classification (in this case, ‘natural’) with no processing, of 82.6% overall accuracy. That the neural networks trained compositional features routinely far exceed the ZeroR benchmark confirms their ability to differentiate natural and transformer-generated sequences.

**Figure 3.**
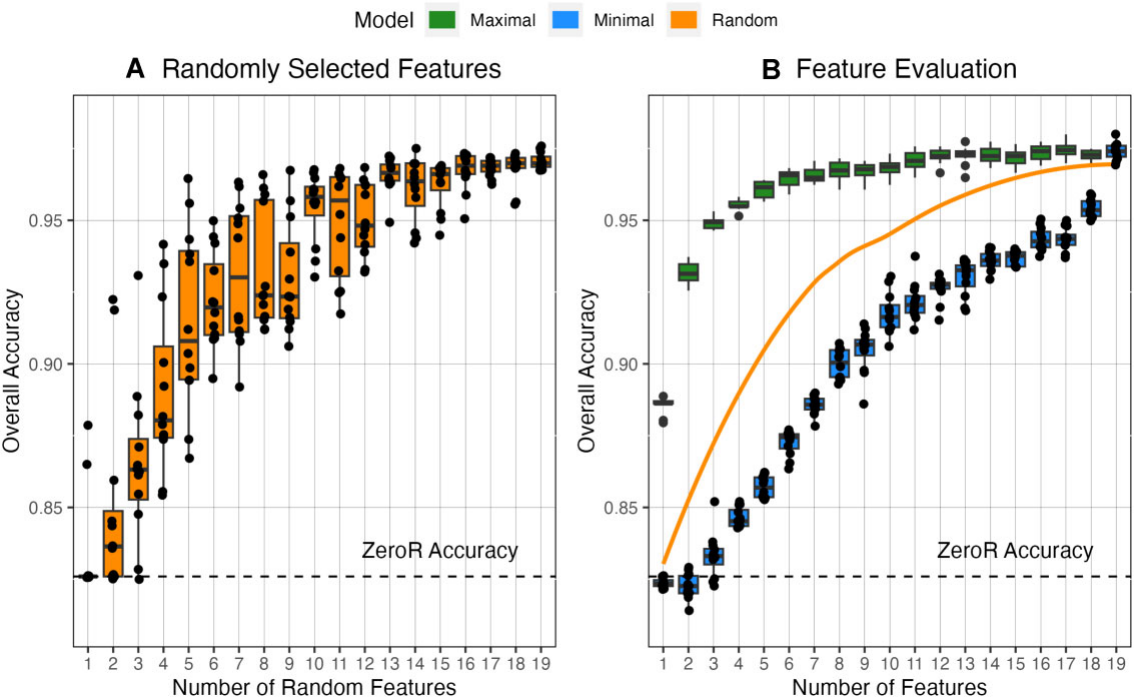
Discrimination of transformer-generated sequences with neural networks trained on varied numbers of features. (**A**) Overall classification accuracy for models built with a random selection of features. Boxplot displays distribution statistics for *n* = 12 replicates. (**B**) Results from predictive feature evaluation (described in detail in the ‘Materials and methods’ section). Feature order for maximal and minimal models available in Supplementary Figure S5. Line displays LOESS regression for random features using the R *stats* package.

To determine whether the neural network classification performance was dependent on the number of total metrics or a specific metric—potentially limiting its generalizability beyond megaDNA—new models were trained on random feature subsets. For each total feature count from 1 to 18, 12 models were trained using a random selection of features (216 models total). Accuracy for these ‘incomplete’ models was assessed using the same test population as for the ‘total’ models. Overall performance was seemingly dependent on the near-full breadth of features, with model performance only reaching 95% of its maximum median accuracy (a threshold of 96.6%) after 16 features were present (Figure 3A).

There was stark variability in the performance of the ‘incomplete’ models that must be explained by which features were included. For example, a model with only five random features displayed an accuracy level of 96.5%, which outperformed exactly half of the models with 14 random features. To determine which metrics were the most and least predictive, new features were iteratively added to baseline models by testing and selecting the metric that led to the greatest—or least—increase in accuracy (Figure 3B; Supplementary Figure S5). Akin to a greedy algorithm, this method selects the locally optimal solution at each step, which may not reflect the ‘true’ global order of feature addition for maximal accuracy.

The results of the predictive feature evaluation were unambiguous as to the importance of MFED in discriminating natural and transformer-generated sequences. Not only was MFED the most predictive single metric (supported by Figure 1D), but its exclusion led to an 18-feature model displaying lower median accuracy than that of a model that only included MFED and GpT, CpC and ApC ratios. Interestingly, for the maximum accuracy feature evaluation, 95% of the improvement from ZeroR to the total model is achieved through the addition of only 8 features (compared to 16 for the random selection). Combined with the significant drop in performance of models not including MFED, this result is suggestive that the performance contribution of dinucleotide ratios is saturated quickly, potentially due to the high correlation rates between dinucleotide ratios (Supplementary Figure S3). Future studies may need to investigate compositional metrics beyond dinucleotide ratios, MFE and MFED values to achieve higher accuracy discrimination.

## Discussion

Effectively leveraging generative AI to overcome problems in biology could lead to breakthroughs across multiple domains. While generative approaches have seen impact in fields such as drug discovery, protein folding and designing functional ribozymes (25–27), the use of transformer models specifically is nascent. In a future state, iterative design pipelines could harness the power of transformer models for generation, complemented by encoder models for functional prediction and evaluation. Therefore, establishing methods to rapidly evaluate the outputs of these early models *in silico* and enable refinement of their architecture and training approaches will advance the development of the field.

As generative AI continues to proliferate, there are legitimate concerns over its misuse. While much of the commentary has focused on using large language models (LLMs) for knowledge discovery and synthesis (28,29), other authors have identified the risk of language models for accelerating the design of biological weapons (BWs) (30). As described by Sandbrink (2023) (30), these ‘biological design tools’ may enable circumvention of known biosecurity measures or BW detection methodologies. While important, discussions around the appropriate oversight of the development of generative AI models for biology are outside the scope of this paper. In general, this author is of the opinion that the risks poised by models that can learn currently unknown functional properties that influence pathogenicity, toxicity and transmissibility of viruses are higher than those of transformer models that can generate new sequences from noise. Studies of the former would be akin to the concerns raised by the experiments conducted by Herfst *et al.*, (2012), where determinants of H5N1 influenza virus airborne transmissibility could be inserted into the backbone of an existing influenza virus (31). This conclusion is also partly informed by the specialist knowledge and equipment as well as the large amount of capital required to synthesize and evaluate a transformer-generated virus in a laboratory setting.

While the notion of transformer-generated pathogens is somewhat fantastical (and the analysis in this paper demonstrates that those capabilities are not available in the near term), there are minor risks of the sequences themselves being used maliciously. For example, fully synthetic sequences could be used to intentionally poison public sequence databases. It is therefore pertinent to be able to discern machine-generated sequences—those that have been manufactured through human intervention—from those arising from natural processes. Similar to the large number of tools available to detect ChatGPT generated text (32), the development of methods that can discern ‘fingerprints’ of transformer models in sequence data should be prioritized.

This paper has demonstrated that these markers exist, at least in the circumstances where labeled data are available. The megaDNA-generated sequences are clearly compositionally distinct from natural bacteriophage genomes, with this finding being consistent regardless of the tested metric (dinucleotide ratios, GC content and secondary structure). Overall, the findings point to two classes of compositional shortcomings in the megaDNA outputs:

1. The distinctness of the megaDNA dinucleotide ratios and GC content (calculated across whole sequences) are suggestive that the model overgeneralizes the distinct compositional biases of its training sequences (Supplementary Figure S4). Rather than generating sequences closely resembling specific families represented in the training set, the model’s outputs appear to be a weighted average of all experienced compositional pressures of its training sequences. It is possible, albeit unlikely, that this issue can be overcome with additional training data. It does, however, suggest that a path forward for the generation of whole genomes that resemble members of existing virus families may require the development of family-specific models. This may also be achieved by prepending taxonomic labels to sequences in the training dataset, as done elsewhere (33). However, the low data availability for the vast majority of viral families will challenge their development in the short term.
2. The dramatic difference in MFED values strongly supports a failure of megaDNA to learn associations between neighboring bases that govern secondary structure (and other nonrandom base distributions). This issue may arise due to MEGABYTE’s impressive long-context capabilities that allow it to reconstruct the spatial organization of a bacteriophage genome [e.g. promoters and genes, as seen in (6)], which also result in it paying less attention to the finer, more localized interactions that underlie these relationships. Resolving this particular issue may require migrating to a different architecture than MEGABYTE.

While this paper was in its final preparations, Zhao *et al.* (2024) preprinted a paper describing GenerRNA, their transformer-based model for the generation of synthetic RNA (34). Based on a configuration similar to the OpenAI GPT-2 architecture, the authors demonstrate that their approach generates short RNAs with realistic secondary structure profiles and protein-binding characteristics. A rapid analysis using natural and generated sequences deposited to their GitHub repository (https://github.com/pfnet-research/GenerRNA) demonstrated that GenerRNA was substantially more similar to its training data than megaDNA sequences, with only two composition metrics having significant differences (Supplementary Figures S7 and S8). Importantly, while GenerRNA sequences did display significantly lower MFED values than their training sequences (as seen for megaDNA), the magnitude of difference was minor (0.070 versus 0.078). The raw MFED value being much greater than 0 is highly suggestive that, unlike megaDNA, the architecture used by GenerRNA has successfully ‘learned’ to maintain the local sequence relationships that underpin RNA secondary structure. This result is suggestive that token-based approaches, which may not be at the single nucleotide resolution, are not inherently flawed at maintaining local context features. Lastly, while the authors do not clarify the token limit of GenerRNA, the 1024 token limit of GPT-2 would be unlikely to recapitulate the genome organization that megaDNA excelled at, so future applications of generating whole genomes will need to balance the pros and cons of each architecture or explore novel methods for combining the two approaches.

The intent of this research was to perform a fair and unbiased assessment of the megaDNA model. While the results focus on potential shortcomings of the current version of the model, its remarkable success in other aspects—such as generating potentially functional promoters and maintaining realistic genome organization—should be celebrated. Bin Shao, the creator of megaDNA, should also be recognized for his willingness to open-source an in-development model.

The findings presented herein are specific to the megaDNA model weights retrieved in late December 2023 and are unlikely to generalize broadly. Increasing the classification performance for these approaches may be achieved by expanding the nucleotide compositional metrics under study to more complex patterns (e.g., trinucleotide ratios), including metrics of amino acid composition for predicted gene products (acknowledging megaDNA is not, at its core, a protein language model), and considering the variability of metrics within component subsequences rather than relying on global metrics as done here. There may also be opportunities to investigate whether transformer-based models are better poised to produce well-conserved proteins across viral species present in training data than those that are under host selection. For future research, the methods and approach described here can be used as a general framework to assess the ability of generative AI to recapitulate the compositional biases inherent to RNA and DNA sequences.

## Supplementary Material

lqae129_Supplemental_File

## Data Availability

All data for this study are available at doi:10.5281/zenodo.11225564. This includes fasta files for the natural and transformer-generated sequences under investigation and cleaned datasets for each figure panel. Natural sequences include their NCBI GenBank accession number. Software to produce the transformer-generated sequences is available at https://github.com/lingxusb/megaDNA.
