# Supplementary material for: Transformer model generated bacteriophage genomes are compositionally distinct from natural sequences
Source: NAR Genom Bioinform. 2024 Sep 18;6(3):lqae129. doi: 10.1093/nargab/lqae129 (PMC11409064; doi:10.1093/nargab/lqae129)
Supplement: lqae129_Supplemental_File [file lqae129_supplemental_file.pdf]

Supplementary Methods

MFED sample size optimization

MFED values are essentially an estimate of the relative ranking of a given MFE value within a pool of MFE values derived from 'similar' sequences. Dinucleotide shuffling returns a single 'similar' sequence that is the product of a valid solution to the Eulerian paths as described in Altschul and Erickson (1985). The shuffled sequence is therefore a single sample of a large population of potential solutions. As MFED values are derived by comparing an input sequence against a selection of shuffled controls, the extent to which that selection represents the characteristics of the population is key to the reproducibility and accuracy of the MFED estimate.

The number of shuffling solutions grows exponentially depending on input sequence length (Figure S6A), and can therefore effectively be treated as infinite for the sequence lengths relevant to this study. Based on the Central Limit Theorem, increased sample sizes will reduce the variability of an MFED estimate compared to other estimates drawn from the same population. However, larger sample sizes also incur higher computational costs and thus a balanced sample size must be selected. To measure the relationship between sample size and MFED estimate variability and identify an optimal sample size, random sequences of length l ranging from 100bp to 400bp were generated and MFED values were processed with varying sample sizes. Values of l included 100bp, 150bp, 200bp, 275bp, and 400bp and 15 replicates were generated for each length.

For each sequence, 15 MFED values were derived based on a shuffling sample size of i. Values of i ranged from 10 to 100 with step sizes of 10. From this population of 15 distinct MFED estimates, the standard deviation s was measured. This process was repeated for 15 total s estimates. The median s value for each value of i was carried forward for further analysis, creating a two column dataframe of i versus median s. The s values were normalized based on the maximum median s value and a linear regression of s vs i was performed. While likely not optimal, a linear equation was used to ensure a monotonically decreasing relationship. The resultant equation describes the number of samples i necessary to achieve a reduction of Y% compared to the maximum s value for that sequence. For every integer value of Y from 1 to 99, the median estimate of i for all input sequences of length l was collected.

GenerRNA Evaluation

This evaluation incorporated the 2000 natural and 2000 generated sequences listed within `MFE_distribution_Fig4a.csv` in the GenerRNA GitHub repository (https://github.com/pfnet-research/GenerRNA) as of commit ab7f470 on January 25, 2024. These were subsetted to only those sequences without ambiguous nucleotide codes, yielding 1,939 natural and 1,942 generated sequences for the analysis. GC content, dinucleotide odds ratios, MFE, and MFED values were calculated for whole sequences (rather than 120bp subsequences for MFE and MFED as done for megaDNA). This approach was selected to be similar to that done in the GenerRNA preprint. MFED was calculated with only 20 iterations to save computational time given size of input sequences. PCA and cluster analysis were performed as in methods.

Gene Functional Evaluation

A subset of .faa files produced by PHANOTATE for 100 transformer-generated sequences were queried against the nr database on NCBI using blastp v2.16.0 by leveraging the Bio.Blast.NCBIWWW module in Biopython.

Supplementary Results

MFED sample size optimization

As expected, the relationship between sample size and MFED estimate variability was logarthmic, with progressively diminishing impacts on variability for increasing sample sizes (Figure S6B). Results were consistent across all sequence lengths tested. From these results, a shuffling sample size of 105 was chosen to be used in this study as this was shown to reduce the value of s by approximately 89% for all sequence lengths. Beyond this level, the diminishing returns of increased sample size were deemed to be an inefficient use of computational resources and time. There is no 'correct' sampling size for dinucleotide shuffling and future uses of this method need to balance accuracy with available resources.

GenerRNA Evaluation

For the 19 compositional metrics analyzed, the distributions of natural versus generated sequences were significantly different for only two after a Bonferroni correction (GpC ratio and MFED, Figure S7). Consistent with the findings of Zhao et al. (2024), there were no differences in distribution of raw MFE values. However, the algorithms used by Zhao et al. (2024) to conclude there were no differences in structure are inadequate compared to dinucleotide shuffling (see Clote et al. (2005)). In contrast to the authors' claims of parity between natural and generated sequences, MFED values of generated sequences were significantly lower than those of natural sequences (0.070 versus 0.078, respectively, $p = 0.000062$, two-tailed Mann-Whitney U Test). Lastly, generated sequences did not cluster with themselves greater than expected by chance ($p = 0.14$, binomial distribution; Figure S8). Compared to megaDNA, the composition of GenerRNA model outputs are substantially more compositionally similar to their training data.

Gene Functional Evaluation

The 100 tested .faa files comprised 6,664 transformer-generated genes predicted by PHANOTATE. Of these, only 11.7% returned at least one hit ($n = 780$). 46.2% of successful queries had > one hit ($n = 360$) for a total of 2,248 hits. The median Expect (E)-value for all hits was a disappointingly high 4.68 (3.81 if queries were limited to only their lowest scoring hit). 153 queries had at least one hit with an E-value of < one, of which only 15 were < 0.05. Exactly three queries had E-values < 0.01. These three hits from three different queries were to a serine/threonine-protein kinase from Pseudomonadota bacterium (Accession = HRI53766; E-value of 0.0027), a rhamnulokinase family protein from ribacterium parvum (Accession = WP_009535588; E-value of 0.0072), and uncharacterized protein LOC131167269 from Malania oleifera (Accession = XP_057982017; E-value of 0.0050). Notably, none of the queries returned hits derived from bacteriophage genes.

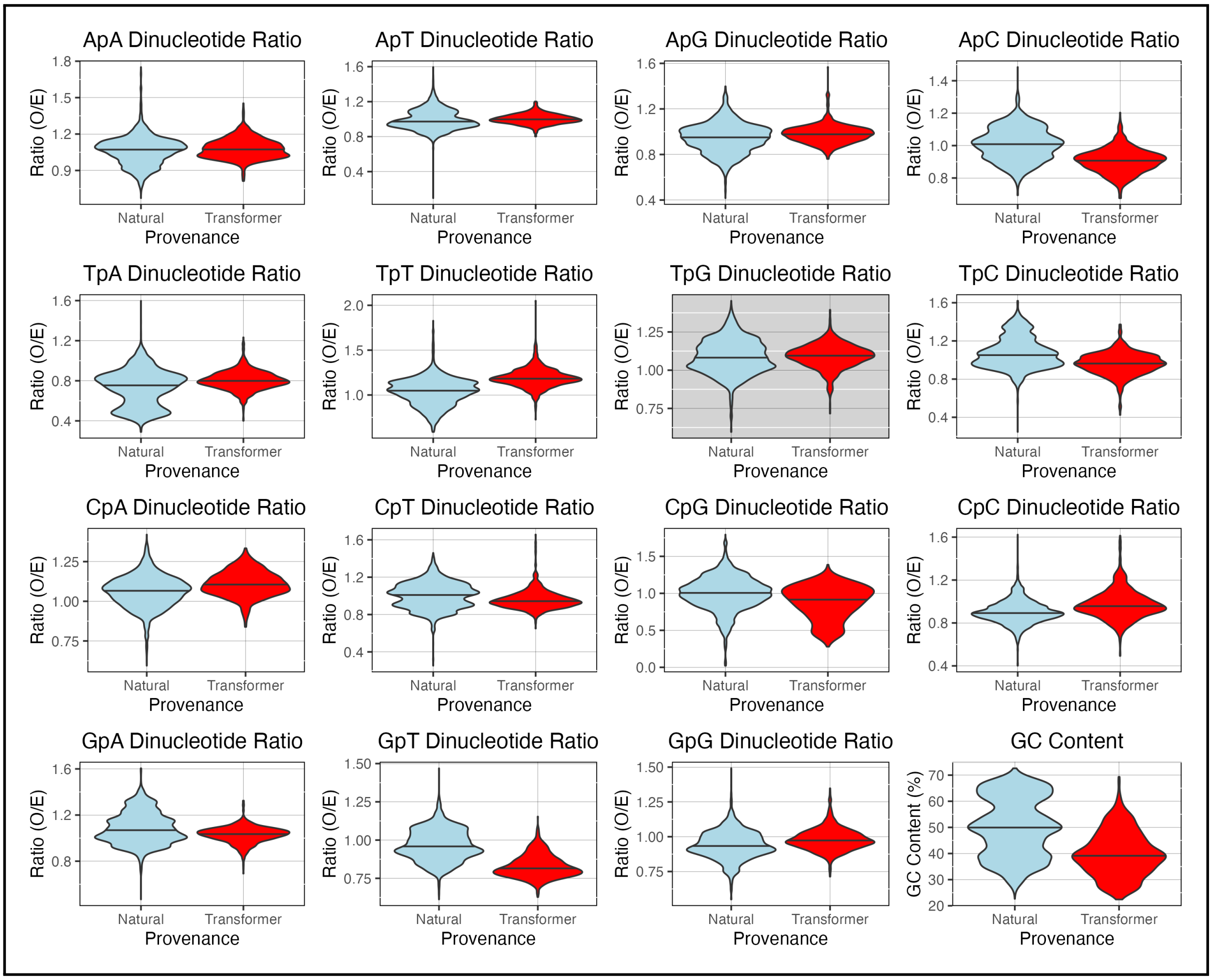

Fig. S1. Comparisons of Compositional Metrics between Natural and Transformer-Generated Sequences.
Inclusive only of metrics not displayed in Figure 1. All distributions significantly different by two-tailed Mann-Whitney U Test ($p < 0.0026$) except for TpG dinucleotide ratio (colored grey).

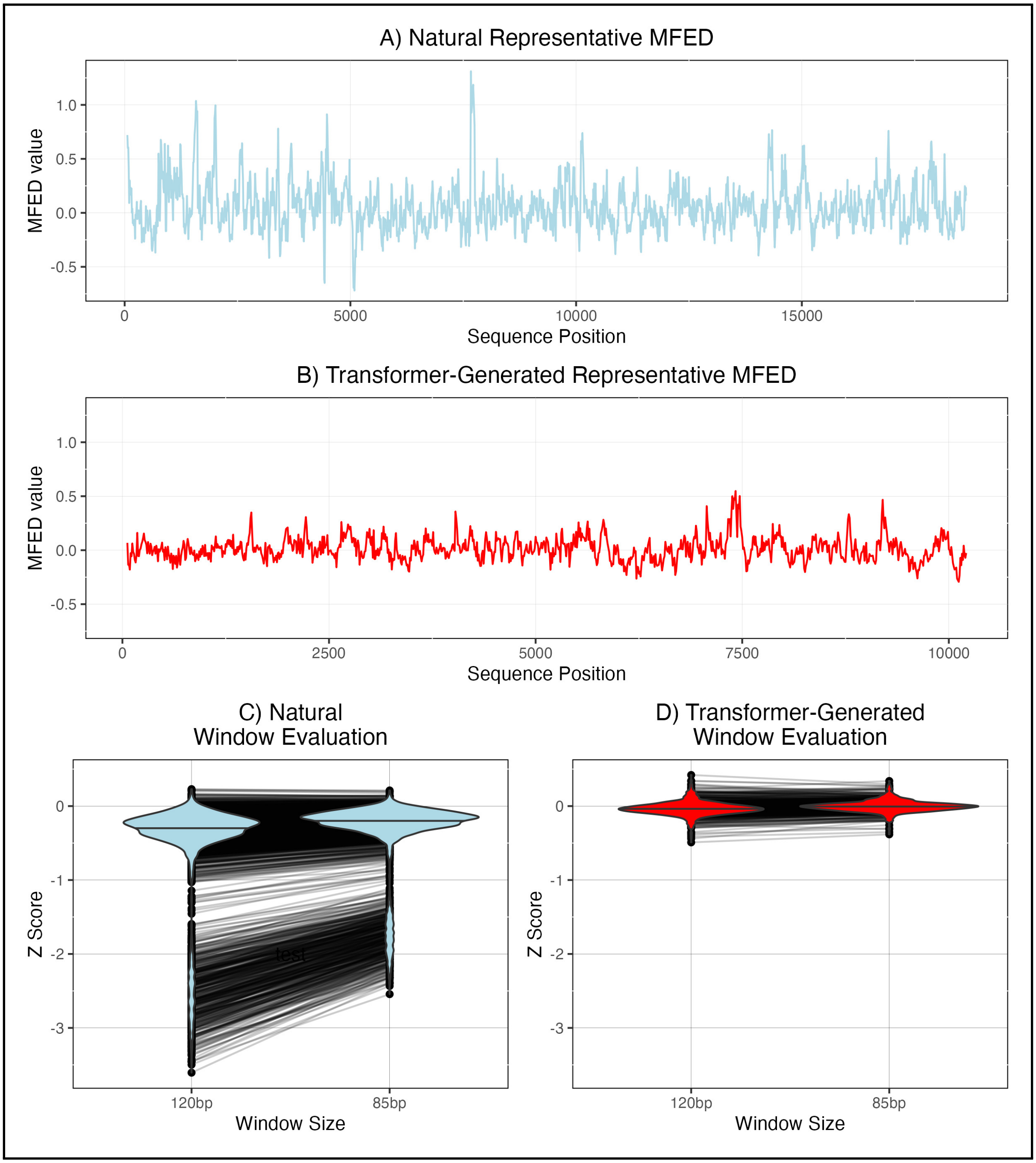

Fig. S2. Exploration of MFED values.

(**A, B**) MFED plots for representative sequences. Sequences chosen due to having MFED values that were the median value for their respective provenance. Note consistent y-axis for both plots. (**C, D**) Impact on median Z score when changing window for MFE/MFED calculation from 120bp to 85 bp. Negative Z scores are indicative of a higher MFED value than expected by chance.

Metric	Natural	Transformer-generated: All	High quality	Medium quality	Low quality
	**A) Median values**				
GC Content	0.499	0.392	0.410	0.384	0.374
ApA ratio	1.074	1.074	1.060	1.065	1.088
ApG ratio	0.952	0.979	0.975	0.984	0.979
ApT ratio	0.973	0.997	1.017	0.995	0.987
ApC ratio	1.010	0.908	0.924	0.908	0.890
GpA ratio	1.070	1.035	1.041	1.041	1.029
GpG ratio	0.933	0.972	0.958	0.967	0.999
GpT ratio	0.958	0.813	0.791	0.812	0.836
GpC ratio	1.040	1.234	1.238	1.239	1.221
TpA ratio	0.756	0.799	0.776	0.800	0.814
TpG ratio	1.082	1.094	1.117	1.103	1.079
TpT ratio	1.050	1.183	1.225	1.187	1.165
TpC ratio	1.055	0.965	0.946	0.955	0.984
CpA ratio	1.068	1.106	1.118	1.113	1.096
CpG ratio	1.005	0.916	0.950	0.896	0.907
CpT ratio	1.013	0.944	0.929	0.948	0.953
CpC ratio	0.893	0.957	0.913	0.960	0.993
MFE	-32.200	-19.700	-21.100	-19.100	-19.000
MFED	0.032	0.005	0.008	0.004	0.004

Metric	Natural vs Transformer	Natural vs High quality	High quality vs Medium quality	High quality vs Low quality	Medium quality vs Low quality
	**B) Comparison**				
GC Content	5.93E-146	8.94E-32	1.77E-06	1.53E-11	0.06
ApA ratio	3.75E-04	0.67	0.21	1.84E-06	8.88E-04
APG ratio	3.32E-15	3.40E-04	0.02	0.23	0.23
ApT ratio	1.12E-10	4.90E-10	1.83E-05	3.99E-09	0.03
ApC ratio	8.57E-188	3.23E-52	4.11E-03	3.52E-09	3.74E-03
GpA ratio	3.17E-30	1.97E-09	0.58	0.03	0.14
GpG ratio	5.57E-38	1.45E-04	5.07E-04	4.39E-13	5.40E-05
GpT ratio	3.55E-294	4.99E-117	4.80E-04	4.70E-10	2.24E-03
GpC ratio	7.99E-212	4.66E-81	0.96	0.04	0.04
TpA ratio	6.22E-31	1.23E-06	1.82E-03	1.16E-05	0.31
TpG ratio	0.07	2.12E-04	9.74E-03	4.91E-09	3.80E-03
TpT ratio	3.65E-217	8.14E-92	5.81E-05	1.37E-16	1.46E-05
TpC ratio	2.42E-94	3.73E-46	0.35	8.97E-06	1.83E-03
CpA ratio	1.05E-46	6.21E-22	0.82	9.88E-04	4.48E-04
CpG ratio	1.04E-44	2.12E-08	2.38E-03	8.67E-04	0.94
CpT ratio	2.81E-30	2.65E-15	0.04	4.43E-04	0.23
CpC ratio	1.73E-62	0.01	5.00E-08	1.72E-23	3.16E-06
MFE	1.06E-229	1.26E-62	3.41E-06	1.01E-08	0.42
MFED	<2.2e-308	1.13E-116	3.77E-06	3.00E-06	0.83

Table S1. Distributions of Composition Metrics.
Table split into two for ease of viewing. (**A**) Comparison of median values for compositional metrics under analysis between natural and transformer-generated sequences. (**B**) Values indicate p value from two-tailed Mann-Whitney U Test. Green highlighted cells indicate comparisons that are statistically significant after Bonferroni correction ($p < 0.0026$ for Natural vs Transformer or $p < 6.6e-4$ for all others).

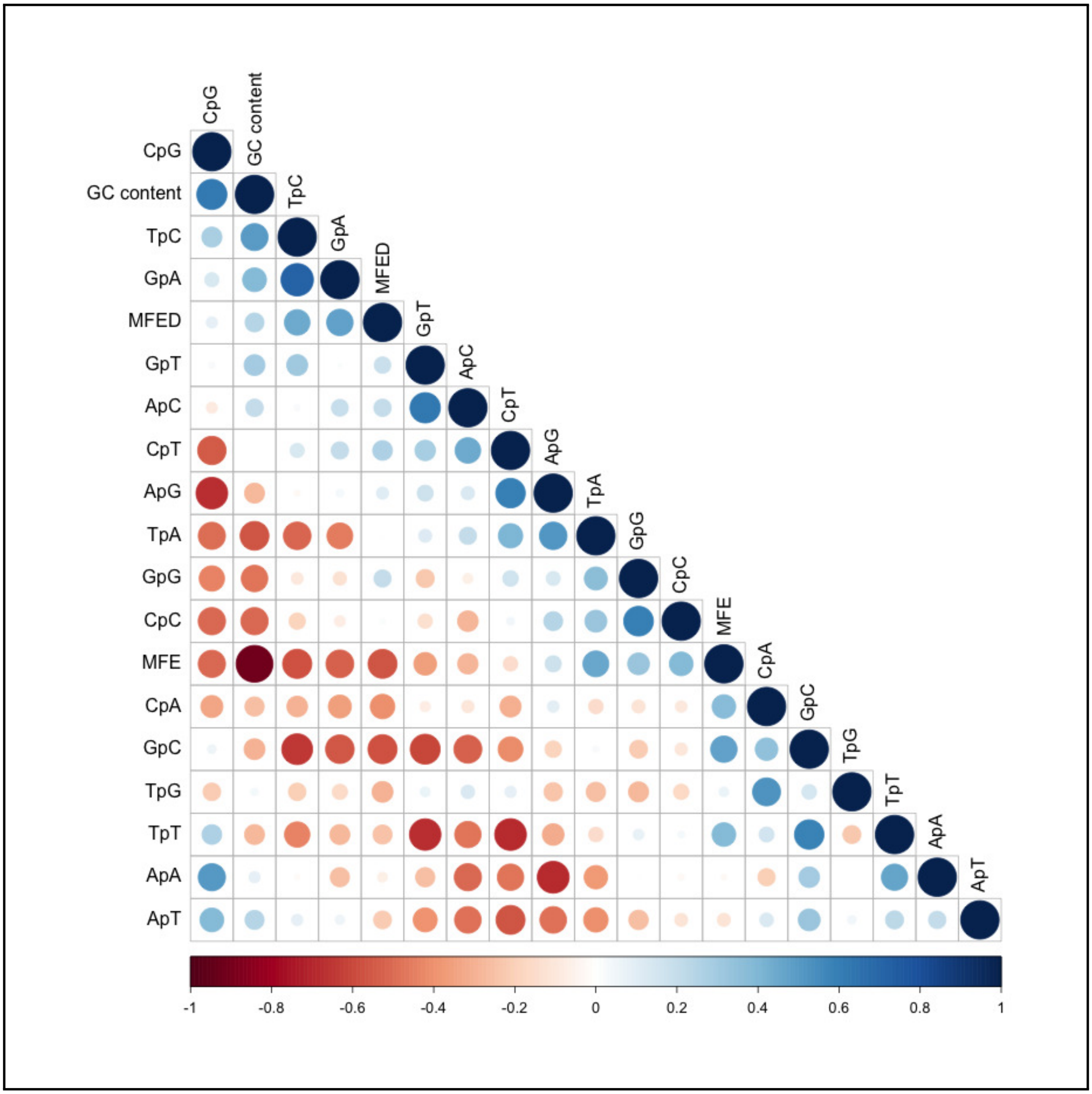

Fig. S3. Correlation between Scaled Compositional Metrics.
Correlations (Pearson) are inclusive of all data points regardless of completeness of taxonomy metadata. Correlation values calculated using function `cor()` from base R.

Family	Nearest Neighbor Matches	Total Members	Frequency Observed	Frequency Expected	P Value
Ackermannviridae	46	50	0.92	0.02	1.41E-80
Aliceevansviridae	93	93	1	0.03	<2.2e-308
Autographiviridae	343	352	0.97	0.11	<2.2e-308
Blumeviridae	5	34	0.15	0.01	1.86E-06
Casjensviridae	44	53	0.83	0.02	3.05E-71
Chaseviridae	29	30	0.97	0.01	4.45E-61
Cystoviridae	14	21	0.67	0.01	1.65E-28
Demerecviridae	84	84	1	0.03	<2.2e-308
Drexlerviridae	105	108	0.97	0.04	2.87E-151
Herelleviridae	94	95	0.99	0.03	2.78E-144
Inoviridae	47	55	0.85	0.02	2.12E-76
Kyanoviridae	35	37	0.95	0.01	2.63E-68
Mesyanzhinovviridae	13	14	0.93	0	1.59E-33
Microviridae	25	30	0.83	0.01	1.31E-48
Orlajensenviridae	11	11	1	0	<2.2e-308
Peduoviridae	84	92	0.91	0.03	1.67E-120
Rountreeviridae	36	38	0.95	0.01	8.70E-70
Salasmaviridae	26	29	0.9	0.01	7.71E-53
Schitoviridae	89	94	0.95	0.03	1.04E-130
Steigviridae	13	14	0.93	0	1.59E-33
Steitzviridae	403	412	0.98	0.13	¡2.2e-308
Straboviridae	151	154	0.98	0.05	1.69E-194
Suoliviridae	33	36	0.92	0.01	1.21E-63
Tectiviridae	7	10	0.7	0	5.50E-19
Vilmaviridae	29	29	1	0.01	<2.2e-308
Zierdtviridae	19	19	1	0.01	<2.2e-308
Zobellviridae	11	12	0.92	0	1.21E-29

Table S2. Family-specific Clustering within PCA Results.
"Nearest Neighbor Matches" defined as the number of family members for which the nearest data point in the 19-dimensional space (by weighted Euclidean distance) was a member of the same family. P value is derived from a binomial distribution where the expectation of matches is equal to the frequency of the family within the broader sample population ("Frequency Expected"). Note, this method is vulnerable to within-family heterogeneity, as a family with many small local clusters but large global variance would appear highly clustered with this method.

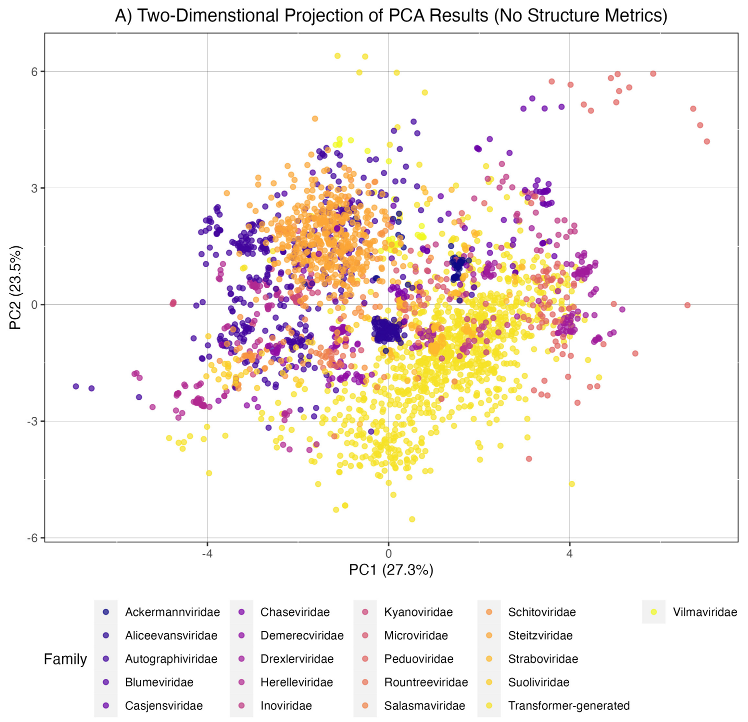

B) Distance from Centroid of Transformer-Generated Sequences (No Structure Metrics)

Family	Euclidean 'Distance'	Total Members
Ackermannviridae	0.56	50
Aliceevansviridae	0.40	93
Autographiviridae	1.10	352
Blumeviridae	0.95	34
Casjensviridae	1.00	53
Chaseviridae	0.65	30
Demerecviridae	0.74	84
Drexlerviridae	0.68	108
Herelleviridae	1.28	95
Inoviridae	0.63	55
Kyanoviridae	1.03	37
Microviridae	0.42	30
Peduoviridae	0.86	92
Rountreeviridae	0.50	38
Salasmaviridae	0.66	29
Schitoviridae	0.94	94
Steitzviridae	1.01	412
Straboviridae	0.21	154
Suoliviridae	1.09	36
Vilmaviridae	0.90	29

Fig. S4. Principal Component Analysis (PCA) of Compositional Metrics without MFE or MFED.
(**A**) Two-dimensional projections of PCs 1 and 2 limited to sequences from families with $\geq$ 25 members, colored by taxonomy. Percentages in x-axis and y-axis titles indicate percentage of variance explaned by given PC. Kindly note that the two-dimensional projection can be misleading as to the true location of a data point in the 19-dimensional space created by the PCA. Transformer-generated sequences clustered at a rate of 92.7% (929/1002). (**B**) Unitless weighted euclidean distance measures from centroid of family to centroid of transformer-generated sequences.

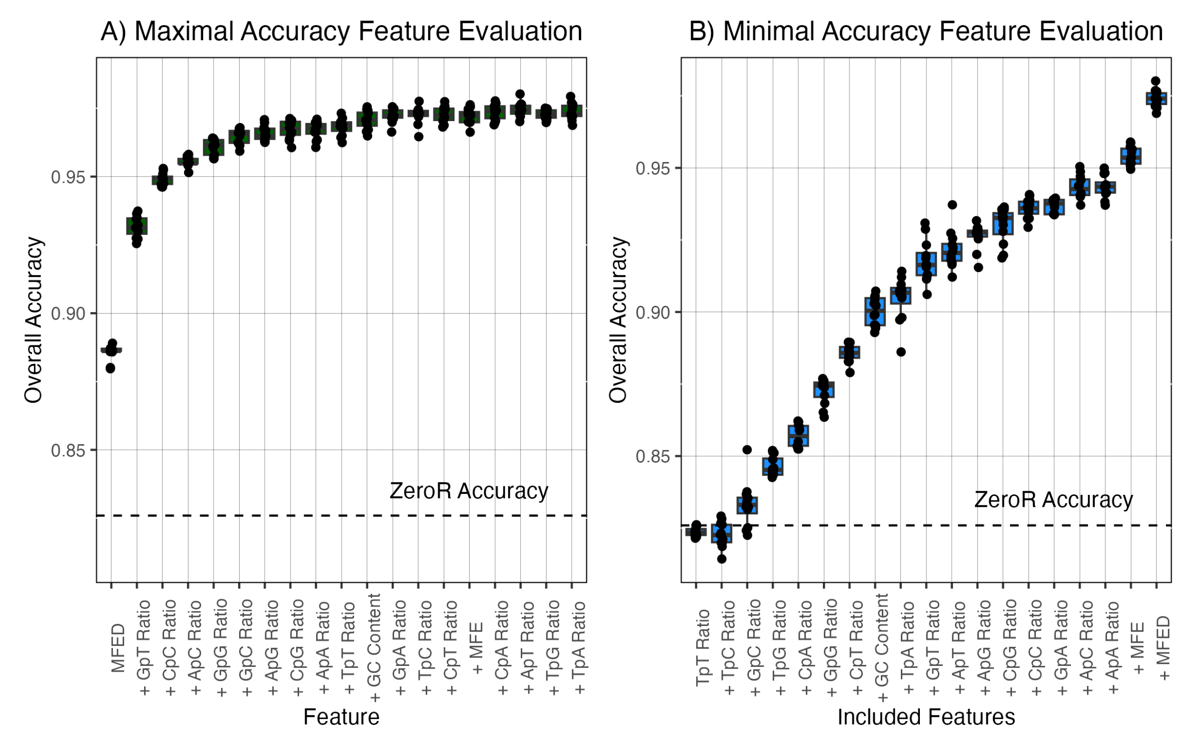

Fig. S5. Neural Network Predictive Feature Evaluation.
Results of predictive feature evaluation for (**A**) maximal and (**B**) minimal feature orders. X-axis labels specify the order in which features were added, with each point on the x-axis specifying the newest feature. Each model was inclusive of all features preceding that point on the x-asix.

(A)

Sequence	Length (bp)	Solutions Observed
CCGATCGATA	10	0
ATCGATCGACCTAGC	15	864
TAGCTAAAGCTGATCGACTC	20	54000
ATCGATCGACTACGAATACACTGAT	25	519878
CGTAGCTCGATCGACTAGGACTGCTAGCCG	30	999691

(B)

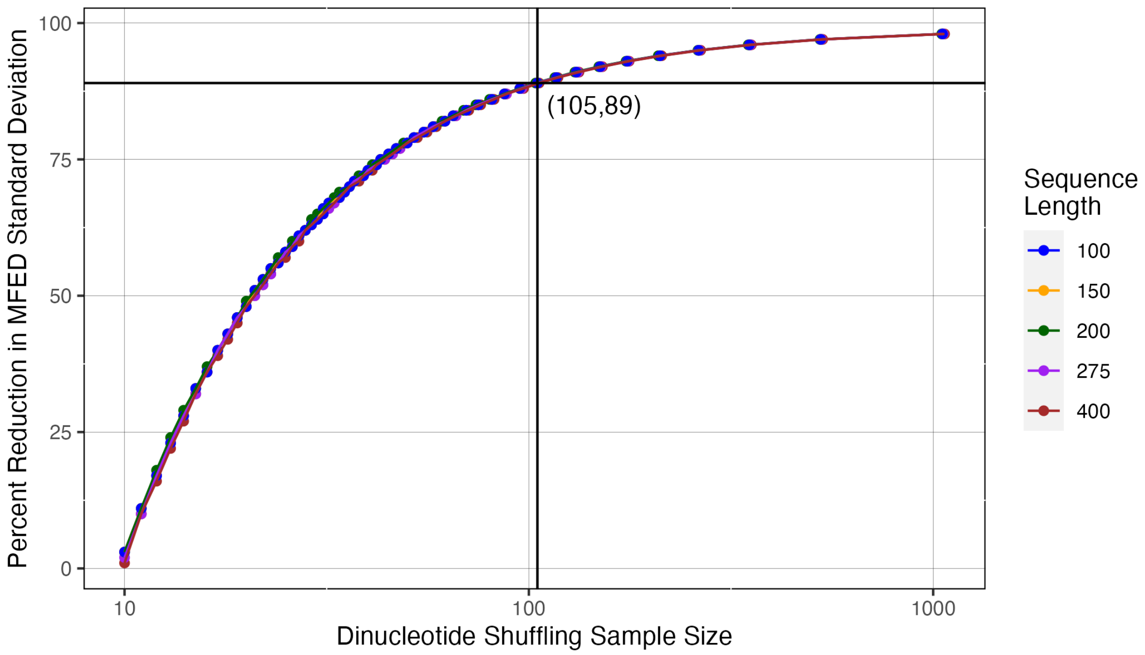

Fig. S6. Dinucleotide Shuffling Sample Size Optimization.
(**A**) Results of dinucleotide shuffling observed for each random input sequence. Solutions observed are number of unique dinucleotide shuffled sequence outputs after performing n = 1,000,000 independent shuffles. (**B**) Relationship between shuffling sample size and variability of MFED estimate. Values obtained as described in Supplementary Methods. Vertex displays sample size implemented for this study.

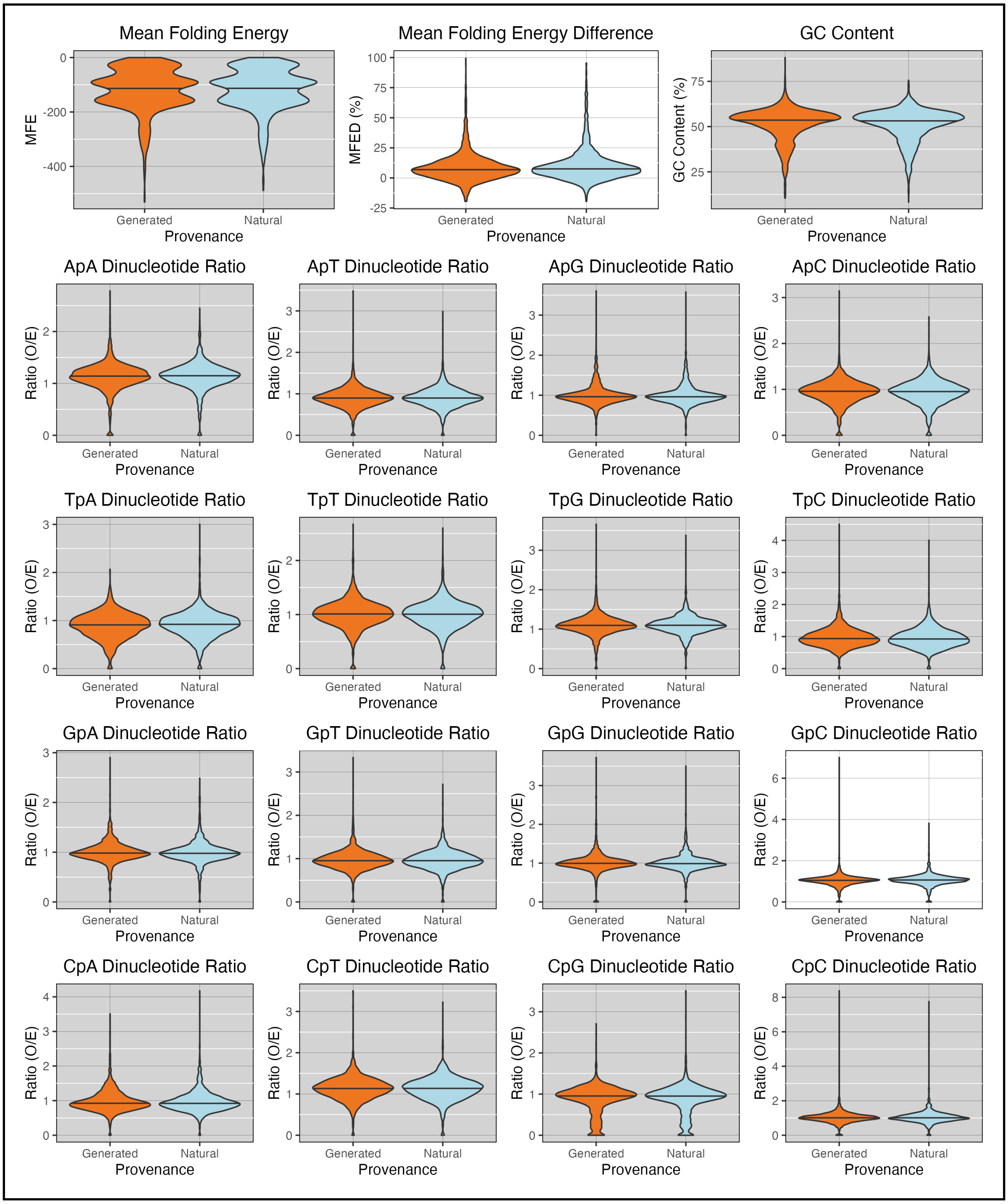

Fig. S7. Comparisons of Compositional Metrics between Natural and GenerRNA Sequences.
Only MFED ($p = 0.00062$) and GpC ratio ($p = 0.0017$) had significantly different distributions by two-tailed Mann-Whitney U Test after a Bonferroni correction. For ease of viewing, these panels are colored white.

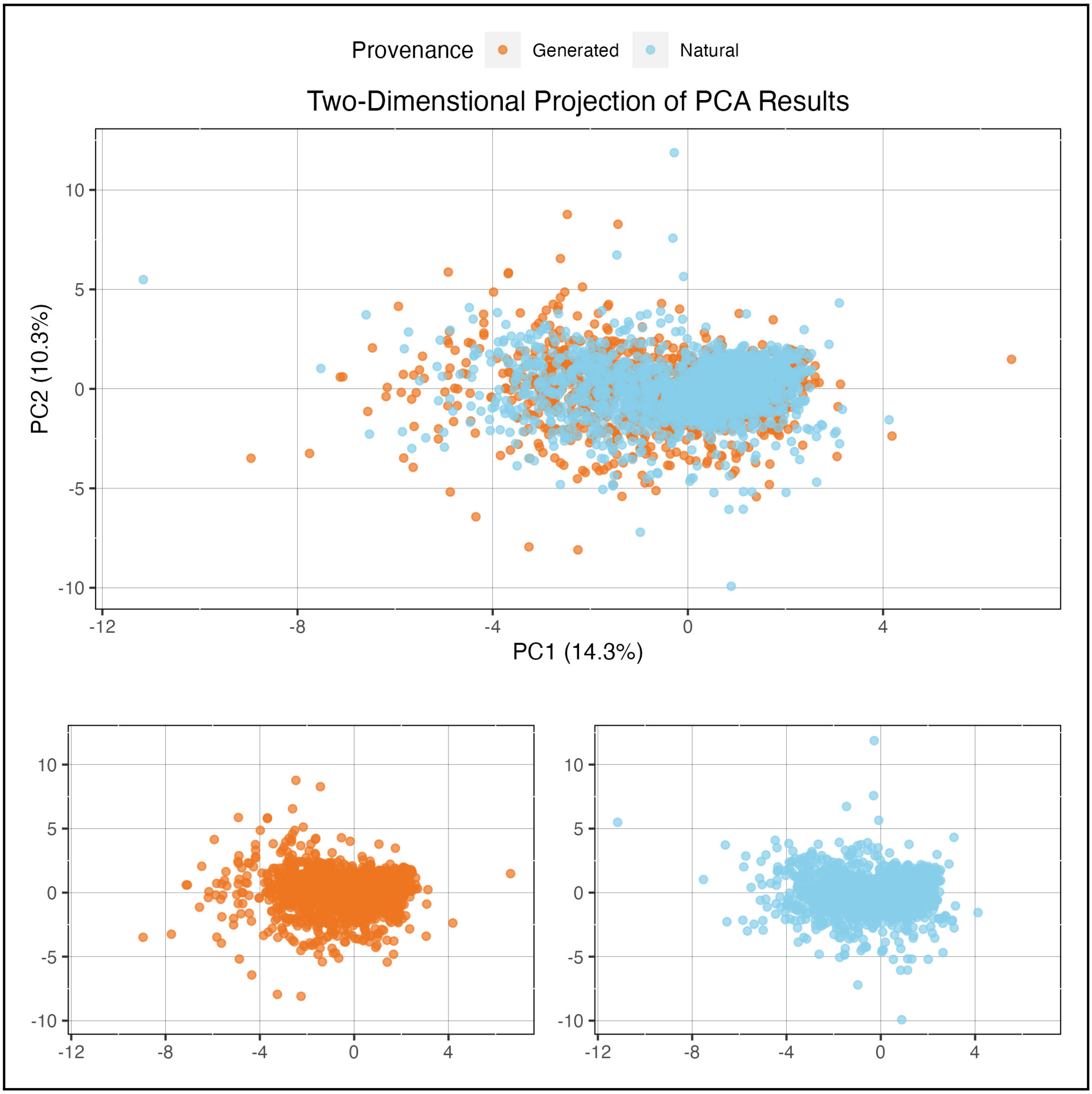

Fig. S8. Principal Component Analysis (PCA) of Compositional Metrics for GenerRNA sequences.
Two-dimensional projection of PCA results for all natural and generated sequences in the GenerRNA dataset. Views of "generated only" and "natural only" provided due to substantial overlap in the main panel. Neither generated nor natural sequences clustered within this analysis by binomial distribution tests.
